# Early ultrasonic vocalization deficits and related thyroarytenoid muscle pathology in the transgenic TgF344-AD rat model of Alzheimer’s disease

**DOI:** 10.3389/fnbeh.2023.1294648

**Published:** 2024-01-23

**Authors:** Denis Michael Rudisch, Maryann N. Krasko, David G. S. Barnett, Kimberly D. Mueller, John A. Russell, Nadine P. Connor, Michelle R. Ciucci

**Affiliations:** ^1^Department of Communication Sciences and Disorders, University of Wisconsin-Madison, Madison, WI, United States; ^2^Department of Surgery, Division of Otolaryngology – Head and Neck Surgery, UW School of Medicine and Public Health, University of Wisconsin-Madison, Madison, WI, United States; ^3^UW Institute for Clinical and Translational Research, UW School of Medicine and Public Health, University of Wisconsin-Madison, Madison, WI, United States; ^4^Wisconsin Alzheimer’s Disease Research Center, School of Medicine and Public Health, University of Wisconsin-Madison, Madison, WI, United States; ^5^Neuroscience Training Program, University of Wisconsin-Madison, Madison, WI, United States

**Keywords:** Alzheimer’s disease, early stage, neurodegenerative diseases, prodromal stage, TgF344-AD rat model, ultrasonic vocalizations, vocal deficits, voice deficits

## Abstract

**Background:**

Alzheimer’s disease (AD) is a progressive neurologic disease and the most common cause of dementia. Classic pathology in AD is characterized by inflammation, abnormal presence of tau protein, and aggregation of β-amyloid that disrupt normal neuronal function and lead to cell death. Deficits in communication also occur during disease progression and significantly reduce health, well-being, and quality of life. Because clinical diagnosis occurs in the mid-stage of the disease, characterizing the prodrome and early stages in humans is currently challenging. To overcome these challenges, we use the validated *TgF344-AD* (F344-Tg(Prp-APP, Prp-PS1)19/Rrrc) transgenic rat model that manifests cognitive, behavioral, and neuropathological dysfunction akin to AD in humans.

**Objectives:**

The overarching goal of our work is to test the central hypothesis that pathology and related behavioral deficits such as communication dysfunction in part manifest in the peripheral nervous system and corresponding target tissues already in the early stages. The primary aims of this study are to test the hypotheses that: (1) changes in ultrasonic vocalizations (USV) occur in the prodromal stage at 6 months of age and worsen at 9 months of age, (2) inflammation as well as AD-related pathology can be found in the thyroarytenoid muscle (TA) at 12 months of age (experimental endpoint tissue harvest), and to (3) demonstrate that the *TgF344-AD* rat model is an appropriate model for preclinical investigations of early AD-related vocal deficits.

**Methods:**

USVs were collected from male *TgF344-AD* (*N* = 19) and wildtype (WT) *Fischer-344* rats (*N* = 19) at 6 months (*N* = 38; WT: *n* = 19; *TgF344-AD*: *n* = 19) and 9 months of age (*N* = 18; WT: *n* = 10; *TgF344-AD*: *n* = 8) and acoustically analyzed for duration, mean power, principal frequency, low frequency, high frequency, peak frequency, and call type. RT-qPCR was used to assay peripheral inflammation and AD-related pathology via gene expressions in the TA muscle of male *TgF344-AD* rats (*n* = 6) and WT rats (*n* = 6) at 12 months of age.

**Results:**

This study revealed a significant reduction in mean power of ultrasonic calls from 6 to 9 months of age and increased peak frequency levels over time in *TgF344-AD* rats compared to WT controls. Additionally, significant downregulation of AD-related genes *Uqcrc2*, *Bace2*, *Serpina3n*, and *Igf2,* as well as downregulation of pro-inflammatory gene *Myd88* was found in the TA muscle of *TgF344-AD* rats at 12 months of age.

**Discussion:**

Our findings demonstrate early and progressive vocal deficits in the *TgF344-AD* rat model. We further provide evidence of dysregulation of AD-pathology-related genes as well as inflammatory genes in the TA muscles of *TgF344-AD* rats in the early stage of the disease, confirming this rat model for early-stage investigations of voice deficits and related pathology.

## 1 Introduction

Alzheimer’s disease (AD) is a progressive neurodegenerative disease and the most common cause of dementia (Tahami Monfared et al., 2022a). Together with Parkinson disease, AD affects over 50 million people worldwide (Goedert, 2015). In 2020, as many as 5.8 million Americans were living with AD and the number is projected to triple by 2060 (Matthews et al., 2019). Between 2000 and 2019 reported deaths from AD increased more than 145% (Alzheimer's Association, 2022). Costs are projected to rise to $1 trillion by 2050, posing tremendous burdens for patients, and caregivers (Schumock, 1998; Matthews et al., 2019; Marasco, 2020; Tahami Monfared et al., 2022b). Seven in ten Americans are hoping to get diagnosed early to allow for timely treatment (Matthews et al., 2019).

AD is generally known for hallmark signs such as progressive memory loss and cognitive dysfunction due to inflammation, abnormal presence of tau protein, and β-amyloid plaques that disrupt normal neuronal function and can lead to apoptotic loss of neurons in frontal and temporal cortex (Goedert, 2015; Jack et al., 2018; Breijyeh and Karaman, 2020; Trejo-Lopez et al., 2022). Recent research suggests that AD is a whole-body disease possibly affecting the peripheral nervous system early in the disease process with early neuronal inflammation and buildup of β-amyloid precursors (Delaby et al., 2015; Jack et al., 2018; Morris et al., 2019; Trushina, 2019; Cheng et al., 2020; Park et al., 2020).

Importantly, vocal deficits and disordered communication also occur early during disease progression, often develop prior to the onset of classic memory and cognitive dysfunction (Garrard et al., 2005; Hajjar et al., 2023), worsen over time, and significantly reduce health and quality of life (Humbert et al., 2010; Mueller et al., 2018a) with devastating associated economic burden (Woodward, 2013). Although signs/symptoms of communication deficits have been traditionally classified as mid-to late-stage events (Ross et al., 1990), recent studies suggest earlier prodromal involvement of other regions in the central and peripheral nervous systems (Delaby et al., 2015; King et al., 2018, 2019; Morris et al., 2019; Trushina, 2019; Cheng et al., 2020; Leuzy et al., 2022a), potentially directly impacting laryngeal coordination and communication (Mueller et al., 2018a; Mahon and Lachman, 2022). Specifically, some of the earliest communication changes in prodromal AD are characterized by subtle changes to temporal speech parameters in individuals who are clinically unimpaired, but show evidence of abnormal, global β-amyloid and/or tau deposition (Mueller et al., 2016, 2018a,b, 2021; Mazzon et al., 2019; Hajjar et al., 2023). Despite recent attention to novel brain (Jack et al., 2018; van Oostveen and de Lange, 2021; Ardanaz et al., 2022; Uchida, 2022; Veitch et al., 2022; Leuzy et al., 2022b), peripheral (Mantzavinos and Alexiou, 2017; Xie et al., 2022), and early behavioral biomarkers (Li et al., 2017; Patel and Masurkar, 2021; Zhang et al., 2022; Hussain et al., 2023), communication often remains overlooked as a potential target for AD detection.

Because diagnosis occurs in mid stage of the disease (Larson et al., 2004; Bradford et al., 2009; de Miranda et al., 2011; Emery, 2011; Rajan et al., 2015; Mantzavinos and Alexiou, 2017; Leuzy et al., 2018; Petersen, 2018; Ausó et al., 2020; Porsteinsson et al., 2021; van Oostveen and de Lange, 2021; Veitch et al., 2022), when laryngeal dysfunction has already progressed (Miguel and Irma, 2020; Mahon and Lachman, 2022; Xiu et al., 2022; Hajjar et al., 2023), quantifying the prodrome and early stages is challenging in humans (Jack et al., 2013; Leuzy et al., 2018; Cummings, 2019). This creates significant gaps in knowledge that limit our understanding for early identification and treatment of AD to improve patient trajectory throughout the disease (The Lancet Neurology, 2017). To overcome these barriers, we propose to map the progression of vocal signs in early AD employing a translational approach (Cohen et al., 2013; Saré et al., 2020; Chaney et al., 2021). Animal models allow for concurrent assessment of behavioral deficits and their biological underpinnings with experimental controls. Rats use ultrasonic vocalizations (USV) as a social means of communication (Barfield et al., 1979; Knutson et al., 2002; Kelm-Nelson et al., 2018; Saito et al., 2019; Krasko et al., 2021) and can modify the behavior of their conspecific partners (Brudzynski and Pniak, 2002; McGinnis and Vakulenko, 2003; Brudzynski, 2005, 2009, 2021; Burgdorf et al., 2007; Johnson et al., 2011). They have been used in several contexts to investigate fine-motor and biological mechanisms underlying the onset and progression of different diseases and associated vocal dysfunction (Ciucci et al., 2008; Basken et al., 2012; Ringel et al., 2013; Grant et al., 2015; Belagodu et al., 2016; Götz et al., 2018; Kelm-Nelson et al., 2018; Caruso et al., 2020; Krasko et al., 2021).

The well-established *TgF344-AD* (F344-Tg(Prp-APP, Prp-PS1)19/Rrrc) rat model has been validated as highly reliable, recapitulating all major hallmark signs such as age-dependent buildup of amyloid-β, tau-pathology, gliosis, and apoptotic loss of neurons (Cohen et al., 2013; Saré et al., 2020; Chaney et al., 2021). It further manifests cognitive, behavioral, and other neuropathological dysfunction akin to AD in humans, including early signs of disease such as prodromal neuroinflammation (Cohen et al., 2013; Saré et al., 2020; Chaney et al., 2021). *TgF344-AD* transgenic rats express human APP with the Swedish mutation (APP K670_M671delinsNL-Swedish) and human PSEN1 with the Δ exon 9 mutation, both driven by the mouse prion promoter (Cohen et al., 2013). Pronuclei of *Fischer-344* zygotes co-injected with two cDNAs encoding human APP and PSEN1 show age-dependent accumulation of amyloid plaques in the hippocampus and cortex, as well as striatum and cerebellum between 6 and 26 months of age, with increased levels of soluble and insoluble Aβ40 and Aβ42. Further, microgliosis, astrogliosis, and learning deficits are apparent by 6 months (Cohen et al., 2013).

It is widely known that peripheral levels of inflammation-related cytokines are correlated with AD pathogenesis and fluctuate during disease progression, however, there is a wide gap in knowledge regarding the link between inflammation and AD (Park et al., 2020). Characterizing dysregulation and abnormal events in the peripheral nervous system is paramount to understanding the mechanisms of AD and its pathogenesis. For example, vocal dysfunction and associated pathology of the thyroarytenoid muscle (TA) with its potential as an early biomarker needs further investigation. Many genes such as *interleukin 1 receptor type 1* (*Il1-r1*) and *innate immune signal transduction adaptor Myd88,* form proinflammatory signaling cascades that are deranged in AD (Zarezadeh Mehrabadi et al., 2022). Anti-inflammatory cytokines such as *interleukin-10* (*Il-10*) reduce inflammation during AD pathogenesis (Park et al., 2020). Increased levels in the central and peripheral nervous systems have been reported in the literature and are linked to disease severity (Strle et al., 2001; Leung et al., 2013). Therefore, upregulation of such genes can serve as an indicator for dysregulation akin to AD. Other AD-related gene expressions such as *protein kinase C delta* (*Pkcδ*) are upregulated in human AD brains and have shown to have a role in amyloid-β processing and activation of the immune system via toll-like receptor signaling (Lucke-Wold et al., 2015; Du et al., 2018). In humans with early-onset AD, studies of their brains have revealed that *ubiquinol-cytochrome c reductase core protein 2 (UQCRC2)*, part of complex III of the electron transport chain, is downregulated contributing to mitochondrial dysfunction (Adav et al., 2019). Furthermore, a reduction in *choline O-acetyltransferase* (CHAT) activity in the central nervous system (CNS) and loss of cholinergic neurons have been associated with AD hallmark pathology and are targets for common pharmacologic interventions (Ferreira-Vieira et al., 2016). It is notable that, while some genes are upregulated in one biomarker, downregulation can occur in other regions, potentially depending on timepoint during disease progression (Park et al., 2020). Monitoring and characterizing pro-inflammatory, anti-inflammatory, and other AD pathology-related gene expressions and their up-/downregulation in corresponding tissues can contribute to understanding the inflammation cascade during different disease stages. Importantly, little is known about inflammatory processes in the larynx.

The overarching goal of this work is to test the central hypothesis that early behavioral changes, such as vocal deficits and underlying muscle pathology, are manifested in the peripheral nervous system already in the early stages of AD and are therefore useful biomarkers for early disease detection. The primary aims of this study are to test the hypotheses that: (1) USV deficits manifest in the prodromal stage (6mo) and progressively worsen in the later stages of AD (9mo), (2) dysregulation in inflammatory gene expressions and AD-related gene expressions are present in the early stages of AD in the TA muscle, and (3) to demonstrate that the *TgF344-AD* rat model is a reasonable model for preclinical investigations of early AD-related vocal deficits and pathology. The TA was chosen as it is one of the primary muscles involved in the production of USVs (Kelm-Nelson et al., 2018; Riede, 2018), and it is affected in other similar diseases in humans (Ma et al., 2020) and rat models (Glass et al., 2019; Lechner et al., 2021; Barnett et al., 2023).

## 2 Methods

### 2.1 *TgF344-AD* rats

All work involving rats was approved by the University of Wisconsin-Madison School of Medicine and Public Health Institutional Animal Care and Use Committee (IACUC) and was conducted in accordance with the National Institutes of Health Guide for the Care and Use of Laboratory Animals (The National Academies Press, 2011).

Male *TgF344-AD* rats (*n* = 19) and male wildtype *Fischer 344* (WT) rats (*n* = 19) arrived from the Rat Resource & Research Center (MO, United States) in 5 different age groups, and were housed in same genotype pairs on a reverse light–dark-cycle (12:12 hours) to allow for testing during the active cycle, with full access to food and water *ad libitum*, as well as standard rodent enrichment (Bigelow et al., 2022). All rats were assigned to testing groups based on their date of birth and testing occurred at 6 and 9 months of age accordingly. The male test rats in this study were sexually naïve at the start of the study. Sexually mature female *Long-Evans* rats (*n* = 8; average age at testing timepoint 1: 3.67 months, SD: 1.75 months; Envigo Research Labs, PA, United States) were used as a stimulus to elicit vocalizations from the male test rats.

Prior to acclimation to USV collection, all rats were habituated to handling and transportation from the vivarium to the testing room for at least 30 min on 3 consecutive days. Acclimation to the testing procedure at each timepoint was performed for 5 days with a one-day break prior to recording. Behavioral acclimation and testing sessions were performed with start times ranging from 9 a.m. to mid-afternoon, under red lighting. To reduce risks for unconscious bias, rats were identified through alphanumeric code numbers, and researchers handling rats, conducting experiments, and analyzing data were blinded to genotype information.

### 2.2 Ultrasonic vocalizations

For vocalization testing, an ultrasonic microphone with 16-bit resolution and sampling rate of 250 kHz (CM16, Avisoft Bioacoustics, Berlin, Germany) attached to an ultrasonic recording system (Avisoft Bioacoustics, Berlin, Germany) was mounted 15 cm above a standard polycarbonate rat home cage to record calls for analysis. Rats were placed in their home cage without their housing-mate. Testing rat calls were elicited by introducing a sexually receptive stimulus female rat into the home cage of the experimental rat. Estrus was confirmed by observing a combination of behavioral signs (e.g., lordosis, ear wiggling, hopping, and darting) (Bialy et al., 2000; Brudzynski, 2021). The stimulus female rat was removed after either 2 mounts by the male rat or following signs of interest and prolonged approach behaviors during the initiation stage (i.e., chasing the female rat, sniffing, genital autogrooming). The recording was started once the stimulus rat was removed and male-only calls were recorded for 5 min. This mating paradigm has been described in previous work (Ciucci et al., 2010; Basken et al., 2012; Johnson et al., 2013; Hoffmeister et al., 2022; Broadfoot et al., 2023; Cullins et al., 2023). All rats in both genotypes displayed a variety of expected behaviors (i.e., exploration and autogrooming) after removal of the stimulus rat, and displayed a variety of call types.

Using *DeepSqueak* software (Coffey, United States), USVs were analyzed for low and high frequency in kHz (as a measure of minimum and maximum frequency of the call contour), peak frequency in kHz (frequency at the greatest amplitude within a call), principal frequency in kHz (median frequency of the frequencies within the call contour), call length (ms), and mean power in dB/Hz (average power spectral density of the call contour without background noise influences) as a measure of call intensity (Coffey et al., 2019). Calls were manually labeled into 16 call types as previously described by Wright et al. (2010).

As shown in Table 1, call types were later collapsed into simple, frequency modulated (FM), and harmonic call categories with short, flat, upward and downward ramp, inverted u, step up and step down calls denoting simple calls; multistep, complex, composite, trill, flat trill combination, and trill with jump calls were marked as frequency modulated (complex) calls; split calls were defined as harmonics, to ensure adequate number of calls for statistical analyses. The number of calls per rat and call type were determined and ratio of complex calls was calculated in % complex (FM, harmonic calls). Ultrasonic vocalizations were collected at 6 months (baseline) and at 9 months of age (early stage) on 3 consecutive days. The day of best performance, based on the total number of calls, was used for each rat for further statistical analysis, described below.

**Table 1:** Overview of grouped call types into simple, frequency modulated (FM), and harmonic call categories.

Simple	FM (Complex)	Harmonic (Complex)
Short	Multistep	Split
Flat	Complex	
Upward Ramp	Composite	
Downward Ramp	Trill	
Inverted U	Flat Trill Combination	
Step Up	Trill with Jumps	
Step Down		

### 2.3 Inflammation in the TA muscle

Reverse transcription-quantitative polymerase chain reaction (RT-qPCR) was used to assay peripheral inflammation and AD-related pathology in the TA muscle. MIQE guidelines were followed in our protocol to ensure reliability and reproducibility of our results (Bustin et al., 2009). Because the testing rats were scheduled to undergo additional testing procedures, we used tissue samples from our separate pilot cohort [male *TgF344-AD* rats (*n* = 6) and male *Fischer-344* WT rats (*n* = 6)], which were harvested at the final 12-month timepoint to characterize peripheral inflammation and AD pathology. All available pilot rats underwent a similar ultrasonic vocalization protocol to the one described.

One hemilarynx of random laterality from each animal was rapidly dissected following decapitation, frozen in Tissue-Tek Optimal Cutting Temperature Compound (Sakura Finetek, Tokyo, Japan) under liquid nitrogen, and stored at −80°C until further processing. Hemilarynges were then dissected (TA muscles) on wet ice under a dissecting microscope, rinsed in ice-cold PBS, and frozen on dry ice until RNA extraction.

Total RNA was extracted from TA muscles using an RNeasy Fibrous Tissue Mini Kit (QIAGEN, Hilden, Germany). RNA quality and concentration were quantified using a NanoDrop ND-1000 UV–Vis spectrophotometer (Thermo Scientific, Waltham, Massachusetts, United States). Three samples with the highest quality and concentration of RNA were chosen from each group (WT*: n* = 3, *TgF344-AD*: *n* = 3). iScript Reverse Transcription Supermix for RT-qPCR (Bio-Rad, Hercules, California, United States) generated cDNA from each sample using 750 ng in addition to no template and no reverse transcriptase negative control reactions.

Primers to genes of interest were designed using Primer-BLAST (National Center for Biotechnology Information, NIH) with ideal annealing temperature at 60°C, spanning an exon-exon junction, and without nonspecific targets (Table 2). Primers were ordered from Integrated DNA Technologies (IDT, Coralville, Iowa, United States). Due to the limited retrievable quantities of RNA from the TA muscle and low expected concentrations of several target mRNAs, samples were first preamplified with SsoAdvanced PreAmp Supermix (Bio-Rad, Hercules, California, United States) using a multi-primer assay cocktail. Following preamplification, reactions were diluted 1:20 in TE buffer except for a pooled sample of cDNA that was diluted 1:5 in TE buffer and used to generate standard curves after 1:10 serial dilution.

**Table 2:** Forward and reverse primer sequences designed using Primer-BLAST with ideal annealing temperature at 60°C, spanning an exon-exon junction without nonspecific targets.

Gene symbol; Gene name	Gene expression target	Forward primer sequence	Reverse primer sequence
**Serpina3n;** serine (or cysteine) peptidase inhibitor, clade A, member 3N	AD specific	5’-CTGATGGTCTCTCAGGTGGTC-3’	5’-GGTAATCGTGCACAGTCCCA-3’
**Uqcrc2;** ubiquinol cytochrome c reductase core protein 2	AD specific	5’-AAAGGGCAACTGCTAGAGCC-3’	5’-TCCCTTGTTGCAGTCACACTTA-3’
**Pkp4;** plakophilin 4	AD specific	5’-GAAGGACCCCAGGGAGTTTG-3’	5’-CACGCAGTTCTCCACTGTCT-3’
**Ctsl;** cathepsin L	AD specific	5’-TCCTGTGAAGAATCAGGGCCA-3’	5’-TCCTCTGAGTCCAGACCTCC-3’
**Bace2;** beta-secretase 2	AD specific	5’–GACTCGCCGAGCCCC-3’	5’-CACAAGAATCCGTACCTTCTGCG-3’
**Prkcδ;** protein kinase C, delta	AD specific	5’–TCCATGCGTAGTGAGGAGGA-3’	5’-TCCCGTTGTTGTCTGGGATG-3’
**Igf2;** insulin-like growth factor 2	AD specific	5’-CCACTTCTGCAGCTCTCCC-3’	5’-ATCCCCATTGGTACCGCAAG-3’
**Il1r1;** interleukin 1 receptor type 1	Pro-inflammatory	5’-CCAAGACCTACGGAGAGGGA-3’	5’-CAGACAGCTGAAGCTTCCCA-3’
**Rorc;** RAR-related orphan receptor C	Pro-inflammatory	5’-CACAGAGACACCACCGAACA-3’	5’-GGCCGAACTTGACAGCATCT-3’
**Jun;** jun proto-oncogene, AP-1 transcription factor subunit	Pro-inflammatory	5’-CCAACCAACGTGAGTGCAAG-3’	5’-GAGGGCATCGTCGTAGAAGG-3’
**Foxp3;** forkhead box P3	Pro-inflammatory	5’-CTGTCAGTCCACTTCACTCAGG-3’	5’-CTTGTCTGAGGCAGGCTGGATA-3’
**Myd88;** MYD88, innate immune signal transduction adaptor	Pro-inflammatory	5’-CTCGCAGTTTGTTGGATGCC-3’	5’-CATGCGACGACACCTTTTCTC-3’
**Il1α;** interleukin 1 alpha	Pro-inflammatory	5’-GGGAGTCAACTCATTGGCG-3’	5’-TGGGTTGGATGGTCTCTTCTAA-3’
**Il1β;** interleukin 1 beta	Pro-inflammatory	5’-GGGCCTCAAGGGGAAGAATC-3’	5’-TTTGGGATCCACACTCTCCAG-3’
**Il6;** interleukin 6	Pro-inflammatory	5’-GCCCACCAGGAACGAAAGTC-3’	5’-TGGCTGGAAGTCTCTTGCGG-3’
**Il12b;** interleukin 12B	Pro-inflammatory	5’-ATCATCAAACCGGACCCACC-3’	5’-ATCTTAGGATCGGCCCCTGC-3’
**Il2;** interleukin 2	Pro-inflammatory	5’-CACTGACGCTTGTCCTCCTT-3’	5’-GTTTCAATTCTGTGGCCTGCT-3’
**Ifnγ;** interferon gamma	Pro-inflammatory	5’-TGTTACTGCCAAGGCACACT-3’	5’-TGTGGGTTGTTCACCTCGAA-3’
**Il10;** interleukin 10	Anti-inflammatory	5’-TTCCCTGGGAGAGAAGCTGA-3’	5’-GACACCTTTGTCTTGGAGCTTA-3’
**Ifnα1;** interferon, alpha 1	Pro/Anti-inflammatory	5’-GGGATGCAACCCTCCTTGAC-3’	5’-CCTCCTTACTCTGTCTTGTCAGC-3’

RT-qPCR with SsoFast EvaGreen supermix (Bio-Rad, Hercules, California, USA) was used with forward and reverse primer final concentrations of 500 nM. 1 μL cDNA was used in each reaction, and all reactions were run in triplicate with a no template and a no reverse transcriptase negative control on each plate. PCR reactions were run and analyzed in the CFX Opus 96 RT-PCR System and CFX Maestro software (Bio-Rad, Hercules, California, USA). Cycle parameters were per supermix protocol with *T*_anneal_ = 60°C melt curve analysis following amplification. Assays shown had PCR reaction efficiencies from 90 to 110%, *R*^2^ > 0.98, and a single product on melt curve analysis. All individual primer assays were run on the same plate with standard curves and values were normalized to the *Hsp90* housekeeping gene (Pfaffl, 2001).

### 2.4 Statistical analyses

#### 2.4.1 Ultrasonic vocalizations

SAS 9.4 (SAS Institute, Inc., Cary, NC) was used for all statistical analyses. A *repeated-measures two-way ANOVA*, with genotype (*TgF344-AD*, WT) and age (6 months, 9 months) as independent variables (each rat was considered the experimental unit) for each call category (simple, FM, harmonic) was used to assess all dependent variables associated with ultrasonic vocalizations (duration, low frequency, high frequency, peak frequency, principal frequency, mean power). All rats were tested at baseline/6 months (*N* = 38; WT: *n* = 19, *TgF344-AD*: *n* = 19). A subset of rats were aged up to the 9-month timepoint (*N* = 18; WT: *n* = 10, *TgF344-AD*: *n* = 8). Number of calls were collected per call type, collapsed into call categories (simple, FM, harmonic), and the number of complex calls were calculated as a %-ratio. Post-hoc analysis was completed using a Tukey–Kramer adjustment. The critical level for significance was set at *α* < 0.05.

#### 2.4.2 RT-qPCR

All reactions were normalized to the *Hsp90* housekeeping gene. A Student’s *t*-test was run on the means of WT (*n* = 3) and AD samples (*n* = 3). The critical level for significance was set at *α* < 0.05. Analysis was performed on *Bio-Rad CFX Maestro* software which accounts for differential reaction efficiencies based on Pfaffl (2001). *Cohen’s d* effect size was calculated using G*Power 3.1.9.7 (Erdfelder, Faul, & Buchner, Düsseldorf, Germany) (Faul et al., 2007).

## 3 Results

### 3.1 Ultrasonic vocalizations

#### 3.1.1 Average call length

**Figure 1:**
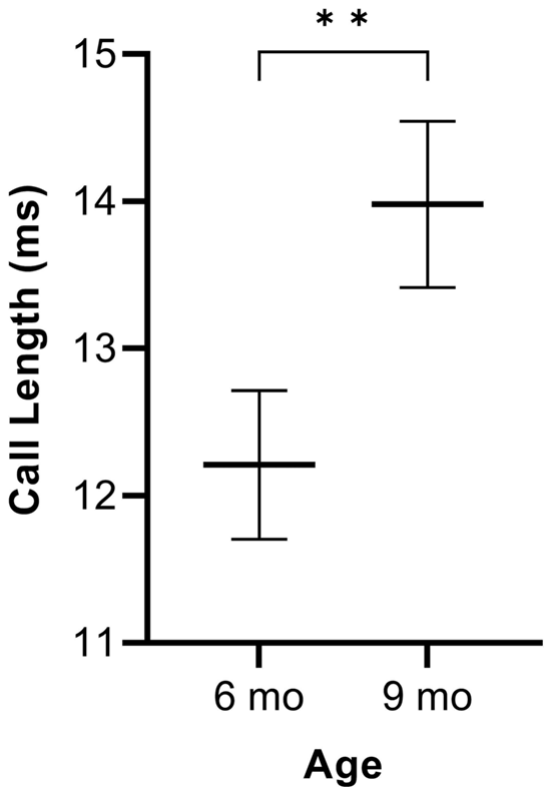
Average call length of simple calls. Data are collapsed across genotype to show the main effect of age. Whiskers indicate standard errors of the means. Lines depict mean call lengths. On average, simple calls are longer at 9 months of age compared to 6 months. **p < 0.01.

In simple calls, there was no significant interaction between genotype and age [*F*(1, 16) = 0.27, *p* = 0.609] for average call length. There was a main effect of age [*F*(1, 16) = 9.19, *p* = 0.008], regardless of genotype [*F*(1, 36) = 3.29, *p* = 0.078]. Average call length for simple calls was significantly longer at 9 months of age than at 6 months (Figure 1). See Table 3 for mean values.

**Table 3:** Least square means estimates (adjusted means) [standard errors (SE)] for acoustic parameters of WT and *TgF344-AD* USVs.

Call Category	Acoustic Parameter	Units	6 months		9 months	
			WT	TgF344-AD	WT	TgF344-AD
Simple	Average principal frequency	kHz	45.035 (0.782)	44.890 (0.749)	43.380 (0.803)	45.870 (0.793)
	Average low frequency	kHz	43.277 (0.769)	43.005 (0.736)	41.490 (0.789)	43.614 (0.780)
	Average high frequency	kHz	46.901 (0.825)	46.937 (0.790)	45.393 (0.846)	48.496 (0.836)
	Average mean power	dB/Hz	−95.057 (0.807)	−94.576 (0.773)	−96.051 (0.828)	−98.230 (0.818)
	Average peak frequency	kHz	45.118 (0.791)	45.130 (0.757)	43.532 (0.812)	46.361 (0.802)
FM	Average principal frequency	kHz	46.665 (0.923)	47.390 (0.904)	46.252 (0.940)	48.3120 (0.940)
	Average mean power	dB/Hz	−92.442 (0.750)	−90.851 (0.722)	−91.614 (0.773)	−93.868 (0.777)
	Average peak frequency	kHz	47.275 (0.870)	48.2555 (0.848)	46.575 (0.889)	49.469 (0.891)

**Figure 2:**
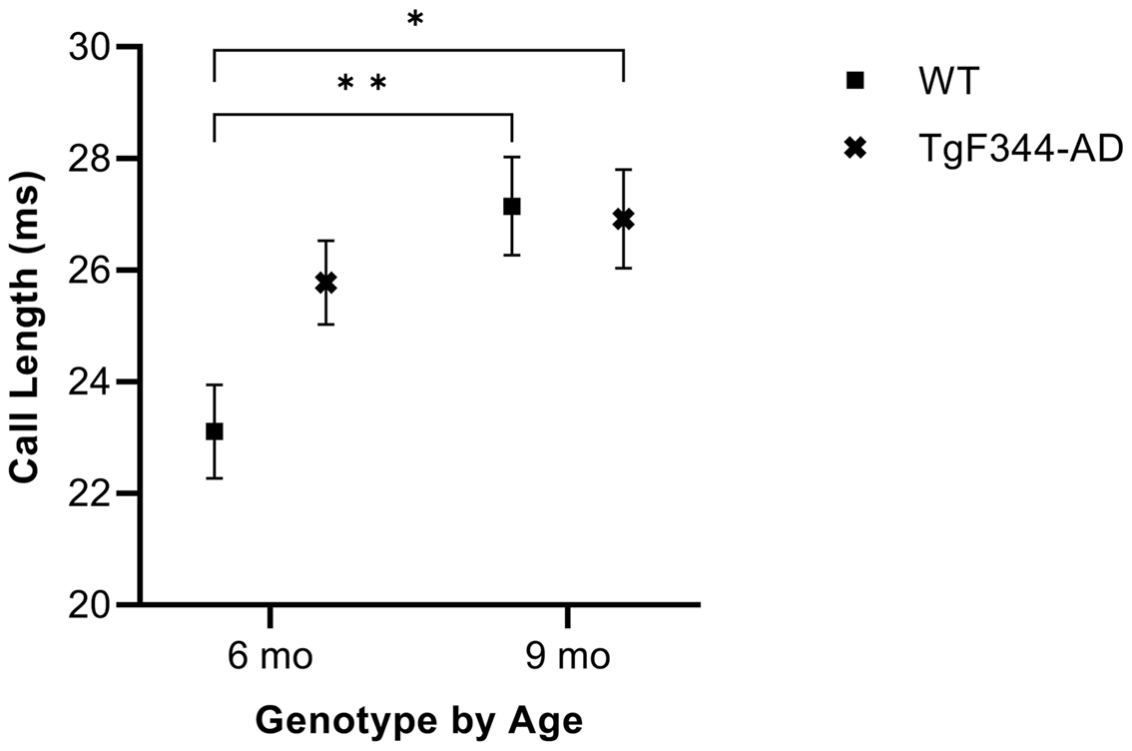
Average call length of FM calls. Squares depict WT group means. Xs show *TgF344-AD* group means. Whiskers indicate standard errors of the means. On average, WT FM calls are shorter at 6 months of age compared to WT FM calls at 9 months. Additionally, WT FM calls are shorter at 6 months of age compared to *TgF344-AD* FM calls at 9 months. *p < 0.05, **p < 0.01.

In FM calls, there was a significant interaction between genotype and timepoint [*F*(1, 14) = 7.31, *p* = 0.017] for average call length. For WTs, average call length for FM calls increased from 6 to 9 months of age (*p* = 0.0018). Additionally, average call length for FM calls was significantly greater for *TgF344-AD* at 9 months than for WTs at 6 months (*p* = 0.033; Figure 2; Table 3).

In harmonic calls, there was no significant interaction between genotype and timepoint [*F*(1, 1) = 1.93, *p* = 0.397] for average call length, nor were there main effects of genotype [*F*(1, 14) = 0.19, *p* = 0.669] or timepoint [*F*(1, 1) = 9.37, *p* = 0.201].

#### 3.1.2 Average principal frequency

**Figure 3:**
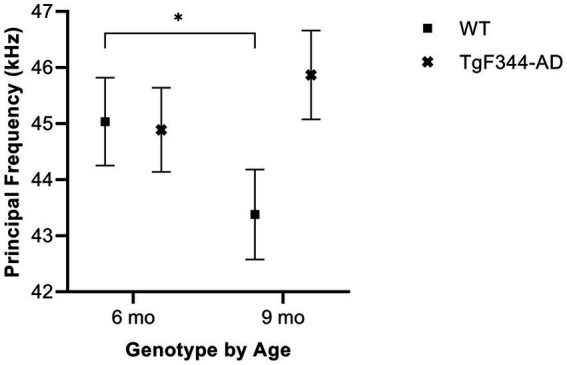
Average principal frequency of simple calls. Squares depict means of WT group calls. Xs show means of *TgF344-AD* group calls. Whiskers indicate standard errors of the means. On average, WT simple calls have a higher principal frequency at 6 months of age compared to WT simple calls at 9 months. *p < 0.05.

In simple calls, there was a significant interaction between genotype and timepoint [*F*(1, 16) = 15.02, *p* = 0.001] for average principal frequency. Specifically, WTs had a greater average principal frequency for simple calls at 6 months than at 9 months of age (*p* = 0.036; Figure 3; Table 3).

In FM calls, there was a significant interaction between genotype and timepoint [*F*(1, 14) = 5.16, *p* = 0.04] for average principal frequency. However, after Tukey–Kramer adjustment, pairwise comparisons revealed no significant differences between groups (*p* > 0.05).

In harmonic calls, there was no significant interaction between genotype and timepoint [*F*(1, 1) = 0.51, *p* = 0.605] for average principal frequency, nor were there main effects of genotype [*F*(1, 14) = 0.00, *p* = 0.972] or timepoint [*F*(1, 1) = 0.04, *p* = 0.875].

#### 3.1.3 Average low frequency

**Figure 4:**
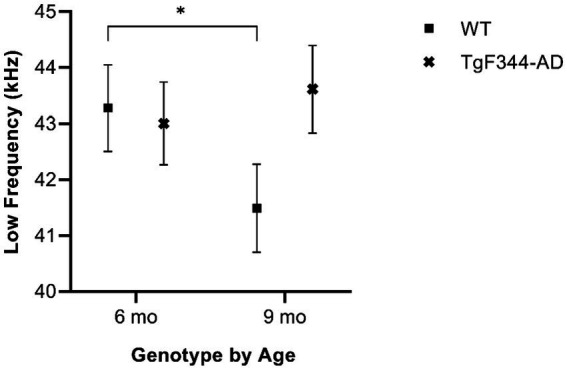
Average low frequency of simple calls. Squares show means of WT group calls, Xs depict means of *TgF344-AD* group calls. Whiskers indicate standard errors of the means. On average, WT simple calls have a higher low frequency at 6 months of age compared to WT simple calls at 9 months. *p < 0.05.

In simple calls, there was a significant interaction between genotype and timepoint [*F*(1, 16) = 12.88, *p* = 0.003] for average low frequency. Specifically, WTs had a higher average low frequency for simple calls at 6 months than at 9 months of age (*p* = 0.02; Figure 4; Table 3).

In FM calls, there was no significant interaction between genotype and timepoint [*F*(1, 14) = 1.90, *p* = 0.19] for average low frequency, nor were there main effects of genotype [*F*(1, 35) = 0.87, *p* = 0.358] or timepoint [*F*(1, 14) = 1.78, *p* = 0.203].

In harmonic calls, there was no significant interaction between genotype and timepoint [*F*(1, 1) = 0.20, *p* = 0.733] for average low frequency, nor were there main effects of genotype [*F*(1, 14) = 4.38, *p* = 0.055] or timepoint [*F*(1, 1) = 12.96, *p* = 0.173].

#### 3.1.4 Average high frequency

**Figure 5:**
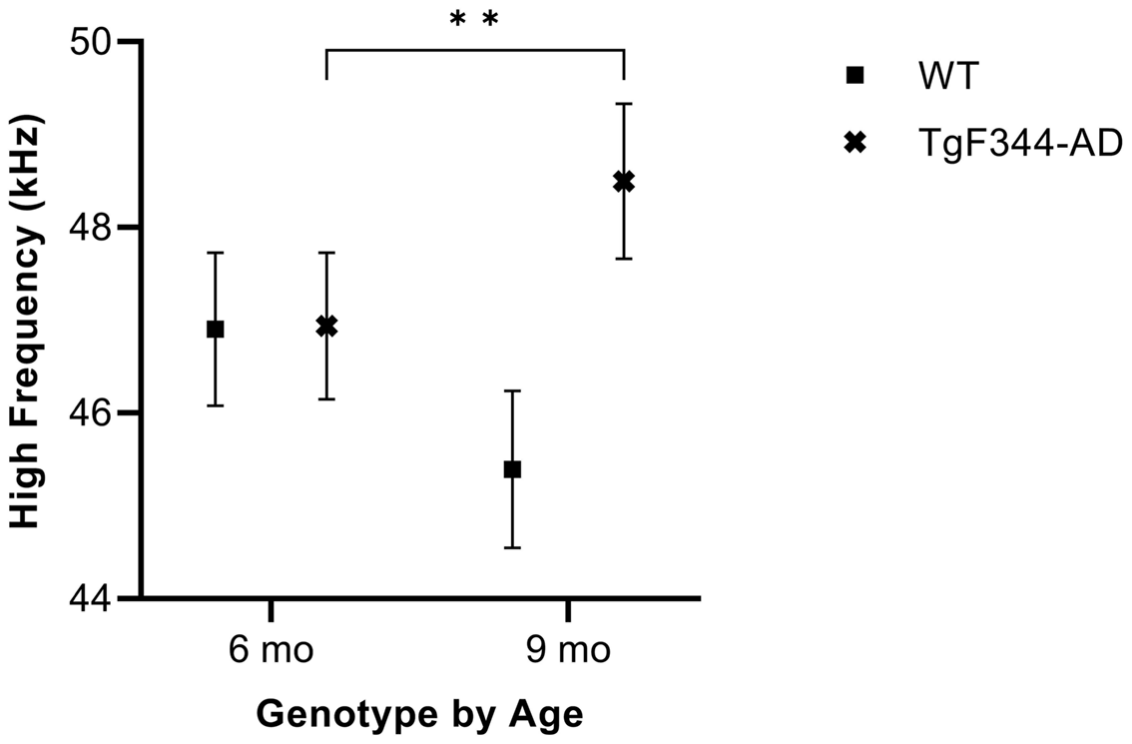
Average high frequency of simple calls. Squares show means of WT group calls. Xs depict means of *TgF344-AD* group calls. Whiskers indicate standard errors of the means. On average, *TgF344-AD* simple calls have a lower high frequency at 6 months of age compared to *TgF344-AD* simple calls at 9 months. **p < 0.01.

In simple calls, there was a significant interaction between genotype and timepoint [*F*(1, 16) = 18.67, *p* = 0.0005] for average high frequency. Specifically, average high frequency for simple calls increased from 6 to 9 months of age in *TgF344-AD* rats (*p* = 0.009; Figure 5; Table 3).

In FM calls, there was no significant interaction between genotype and timepoint [*F*(1, 14) = 2.87, *p* = 0.113] for average high frequency, nor were there main effects of genotype [*F*(1, 35) = 2.91, *p* = 0.1] or timepoint [*F*(1, 14) = 1.87, *p* = 0.193].

In harmonic calls, there was no significant interaction between genotype and timepoint [*F*(1, 1) = 0.09, *p* = 0.816] for average high frequency, nor were there main effects of genotype [*F*(1, 14) = 0.00, *p* = 0.955] or timepoint [*F*(1, 1) = 0.24, *p* = 0.712].

#### 3.1.5 Average mean power

**Figure 6:**
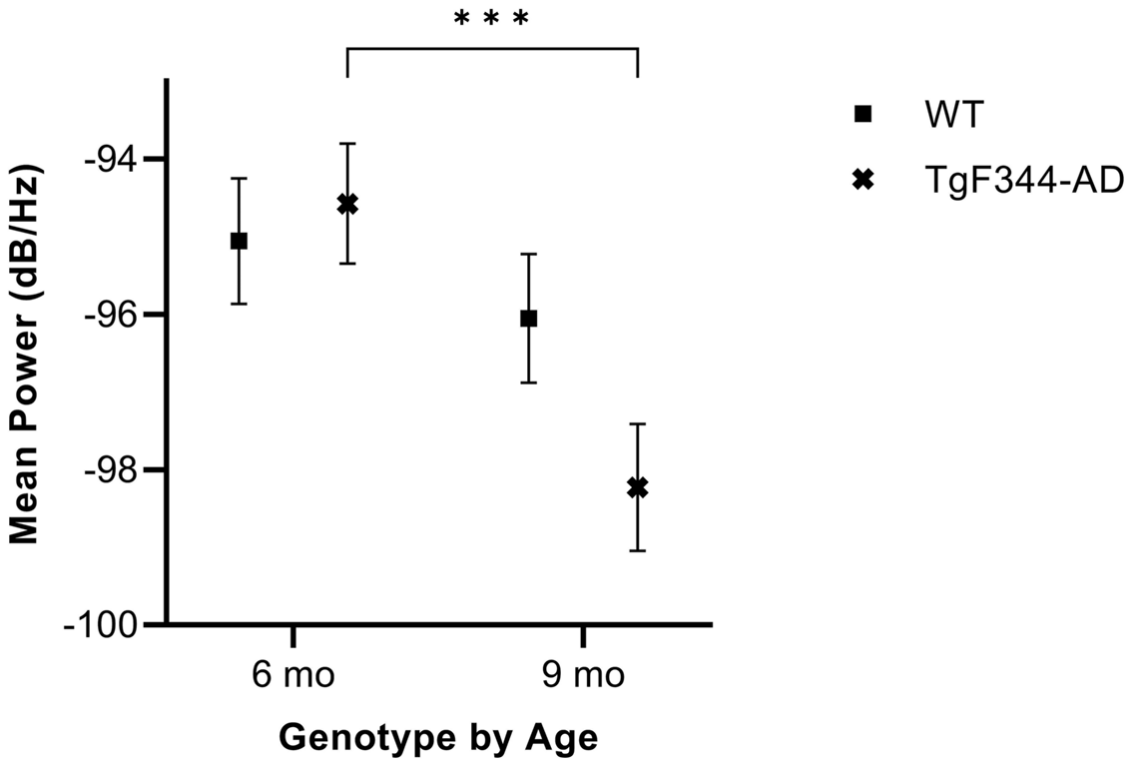
Average mean power of simple calls. Squares show means of WT group calls. Xs depict means of *TgF344-AD* group calls. Whiskers indicate standard errors of the means. On average, *TgF344-AD* simple calls have a higher mean power at 6 months of age compared to *TgF344-AD* simple calls at 9 months. ***p < 0.001.

In simple calls, there was a significant interaction between genotype and timepoint [*F*(1, 16) = 14.59, *p* = 0.002] for average mean power. Specifically, average mean power for simple calls decreased from 6 to 9 months of age in *TgF344-AD* rats (*p* < 0.0001; Figure 6; Table 3).

**Figure 7:**
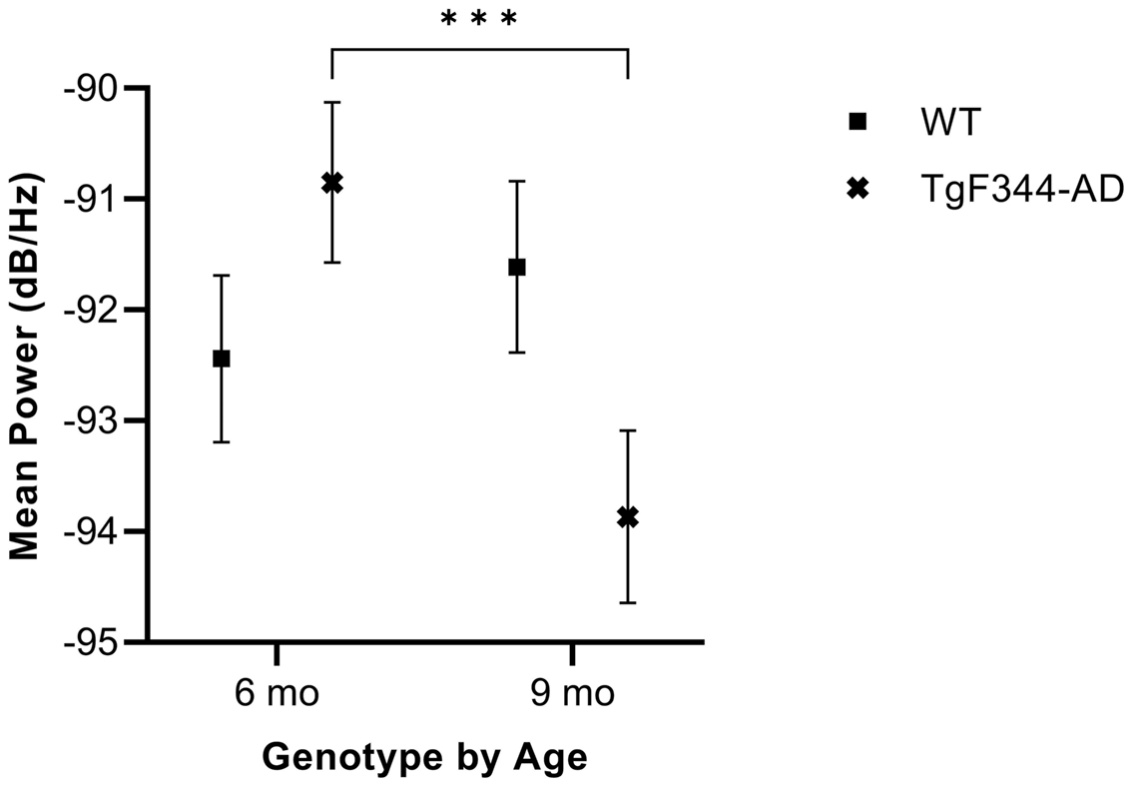
Average mean power of FM calls. Squares show means of WT group calls. Xs depict means of *TgF344-AD* group calls. Whiskers indicate standard errors of the means. On average, *TgF344-AD* FM calls have a higher mean power at 6 months of age compared to *TgF344-AD* FM calls at 9 months. ***p < 0.001.

In FM calls, there was a significant interaction between genotype and timepoint [*F*(1, 14) = 34.06, *p* < 0.0001] for average mean power. Specifically, average mean power for FM calls decreased from 6 to 9 months of age in *TgF344-AD* rats (*p* < 0.0001; Figure 7; Table 3).

In harmonic calls, there was no significant interaction between genotype and timepoint [*F*(1, 1) = 0.26, *p* = 0.701] for average mean power, nor were there main effects of genotype [*F*(1, 14) = 0.00, *p* = 0.98] or timepoint [*F*(1, 1) = 0.02, *p* = 0.916].

#### 3.1.6 Average peak frequency

**Figure 8:**
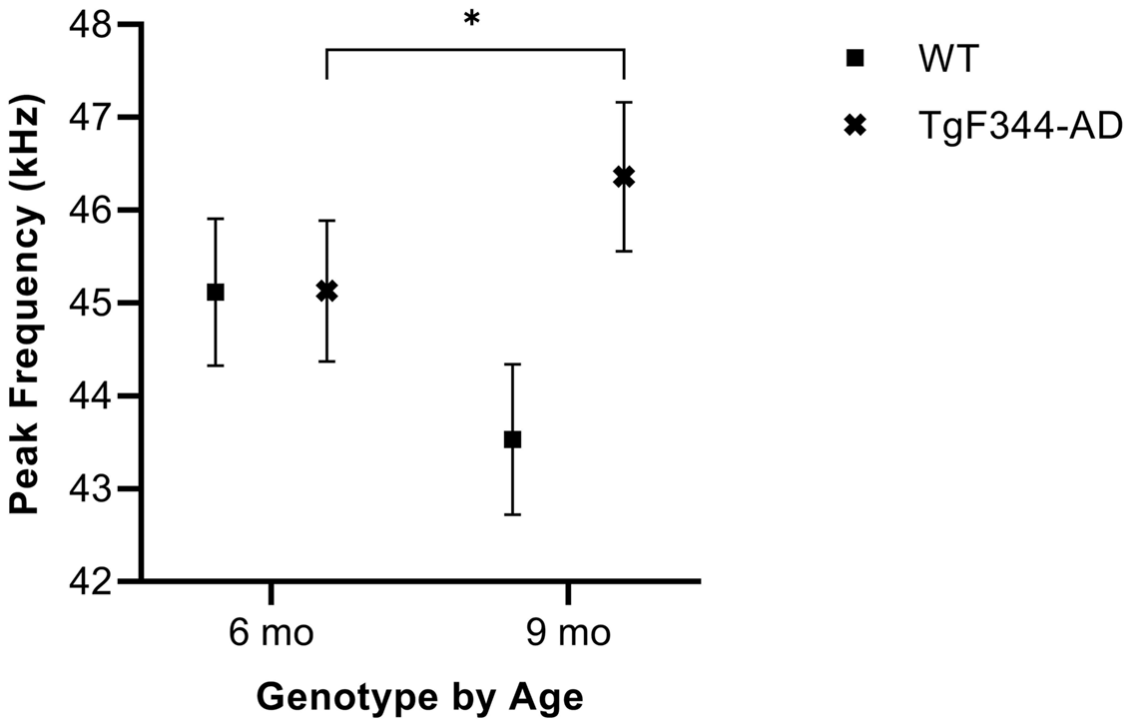
Average peak frequency of simple calls. Squares show means of WT group calls. Xs depict means of *TgF344-AD* group calls. Whiskers indicate standard errors of the means. On average, *TgF344-AD* simple calls have a lower peak frequency at 6 months of age compared to *TgF344-AD* simple calls at 9 months. *p < 0.05.

In simple calls, there was a significant interaction between genotype and timepoint [*F*(1, 16) = 16.71, *p* = 0.0009] for average peak frequency. Specifically, average peak frequency for simple calls increased from 6 to 9 months of age in *TgF344-AD* rats (*p* = 0.04; Figure 8; Table 3).

**Figure 9:**
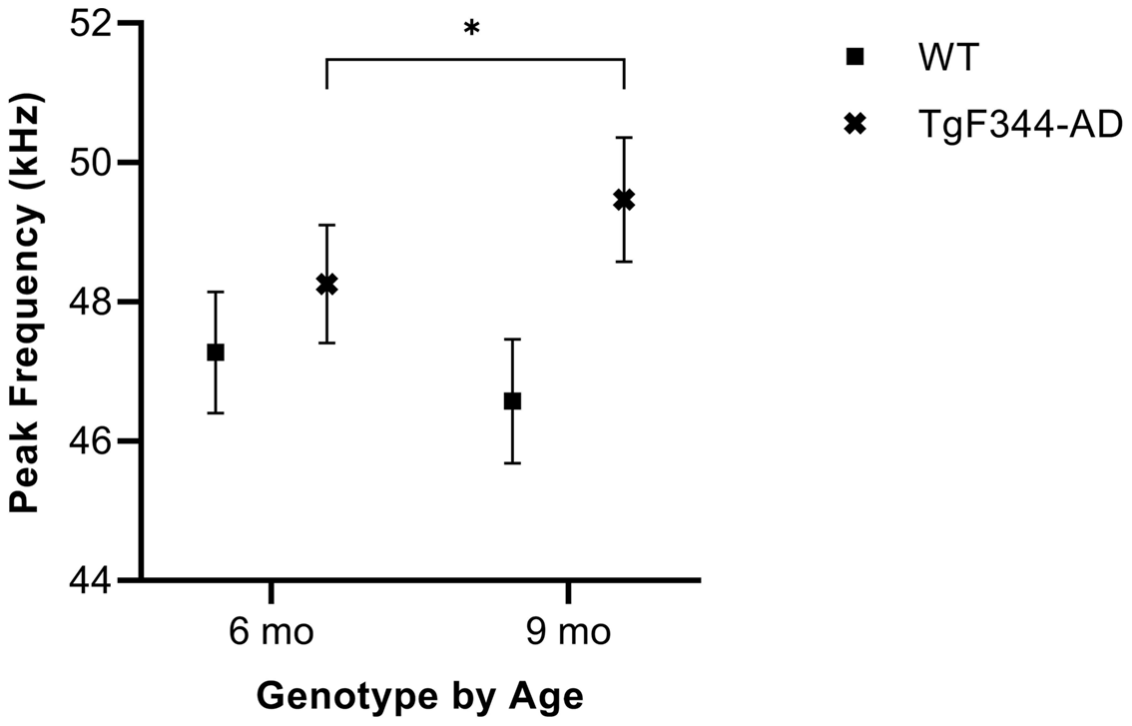
Average peak frequency of FM calls. Squares show means of WT group calls. Xs depict means of *TgF344-AD* group calls. Whiskers indicate standard errors of the means. On average, *TgF344-AD* FM calls have a lower peak frequency at 6 months of age compared *to TgF344-AD* FM calls at 9 months. *p < 0.05.

In FM calls, there was a significant interaction between genotype and timepoint [*F*(1, 14) = 9.17, *p* = 0.009] for average peak frequency. Specifically, average peak frequency for FM calls increased from 6 to 9 months of age in *TgF344-AD* rats (*p* = 0.02; Figure 9; Table 3).

In harmonic calls, there was no significant interaction between genotype and timepoint [*F*(1, 1) = 0.18, *p* = 0.742] for average peak frequency, nor were there main effects of genotype [*F*(1, 14) = 0.16, *p* = 0.692] or timepoint [*F*(1, 1) = 2.58, *p* = 0.354].

#### 3.1.7 Percent complex calls

There was no significant interaction between genotype and timepoint [*F*(1, 16) = 1.58, *p* = 0.226] for percent complex calls, nor were there main effects of genotype [*F*(1, 36) = 0.19, *p* = 0.665] or timepoint [*F*(1, 16) = 4.33, *p* = 0.054].

### 3.2 AD-pathology and inflammation in the TA muscle

**Figure 10:**
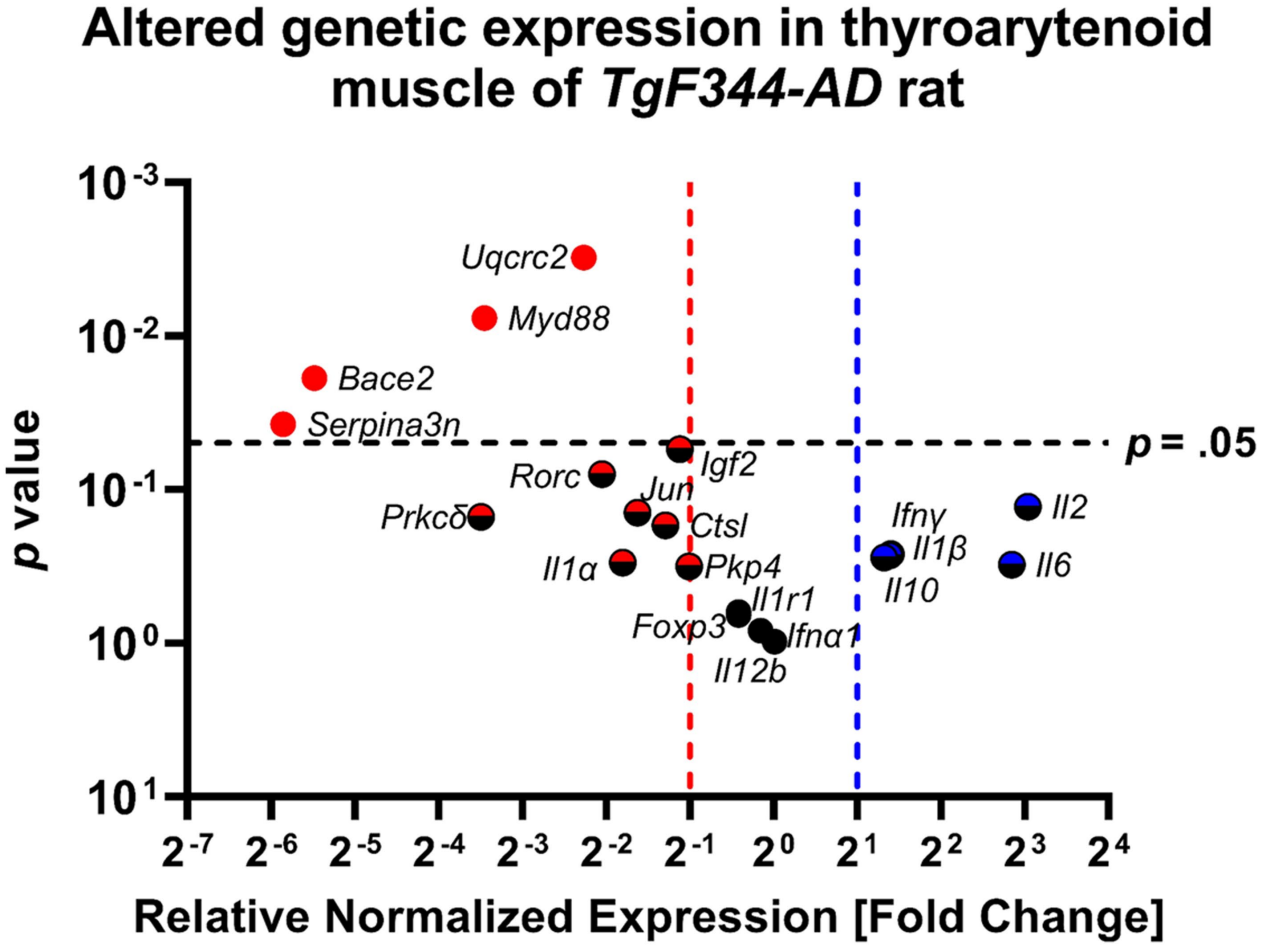
The thyroarytenoid muscle has significantly downregulated genes associated with AD and inflammation. Data represent PCR reactions run in triplicate with *n = 3* WT and *n = 3* AD samples, normalized to *Hsp90* housekeeping gene expression, and AD relative to WT values (∆∆Cq). The differences between the groups are plotted on the x-axis, the *p*-value of the differences are plotted on the y-axis. Each circle in this Volcano Plot represents a specific gene target. Black indicates no significance; blue and red indicate upregulation and downregulation respectively, > 2-fold. Points to the right of the blue dashed line are denoted with a blue or blue/black icon and are upregulated, ≥ 2-fold. Points to the left of the red dashed line are denoted with a red or red/black icon and are downregulated. Points above the horizontal black dashed line have a *student’s t-*test *p*-value ≤0.05 and are denoted with a solid-colored icon.

Table 4 provides an overview of fold change values and corresponding *p*-values for all AD-related genes as well as pro-and anti-inflammatory markers. Figure 10 illustrates altered gene expressions in the TA muscle of the *TgF344-AD* rat.

**Table 4:** Gene targets and their relative normalized expression and corresponding *p*-values.

Gene	Relative normalized expression [fold-change (FC)]	*p-*value
*Uqcrc2*	0.20814	0.003079**
*Myd88*	0.0913	0.007661**
*Bace2*	0.02239	0.018825*
*Serpina3n*	0.01724	0.037509*
*Igf2*	0.46127	0.054402
*Rorc*	0.24166	0.079437
*Il2*	8.21101	0.129354
*Jun*	0.32406	0.14155
*Prkcδ*	0.08886	0.150646
*Ctsl*	0.4091	0.170998
*Ifnγ*	2.6413	0.262451
*Il1β*	2.61231	0.268477
*Il10*	2.50188	0.276235
*Il1α*	0.28678	0.298259
*Il6*	7.18687	0.307731
*Pkp4*	0.49673	0.315929
*Il1r1*	0.7495	0.627926
*Foxp3*	0.74995	0.657188
*Ifnα1*	0.90013	0.829271
*Il12b*	1.00784	0.976202

Asterisks indicate significance. **p < 0.01, *p < 0.05.

#### 3.2.1 AD-related genes

Significant downregulation of AD-related genes in the *TgF344-AD* rat at 12 months of age was found in: *Uqcrc2* (FC = 0.208; *p* = 0.003; *d* = 6.712), *Bace2* (FC = 0.022; *p* = 0.019; *d* = 3.117), *Serpina3n* (FC = 0.017; *p* = 0.038; *d* = 2.502), and *Igf2* (FC = 0.461; *p* = 0.054; *d* = 2.200).

Further downregulation was found in *Pkp4* (FC = 0.497; *p* = 0.316; *d* = 0.935), *Ctsl* (FC = 0.409; *p* = 0.171; *d* =  1.360), and *Prkcδ* (FC = 0.089; *p* = 0.151; *d* = 1.449), however, *p*-values were not within the significance threshold. With large *Cohen’s d* effect sizes, it is likely that undersampling is leading to a lack of significance.

#### 3.2.2 Pro-inflammatory markers

Significant downregulation of pro-inflammatory genes in *TgF344-AD* rats at 12 months of age were found only in *Myd88* (FC = 0.091; *p* = 0.008; *d* = 2.399).

Further downregulation was found in *Rorc* (FC = 0.242; *p* = 0.079; *d* = 2.572), *Jun* (FC = 0.324; *p* = 0.142; *d* = 1.493), and *Il1α* (FC = 0.287; *p* = 0.298; *d* = 0.975) without significance, however, with large *Cohen’s d* effect sizes.

The following pro-inflammatory genes were upregulated without statistical significance, but with large *Cohen’s d* effect sizes: *Il2* (FC = 8.211; *p* = 0.129; *d* = 1.556), *Il6* (FC = 7.187; *p* = 0.308; *d* = 0.954), *Ifnγ* (FC = 2.641, *p* = 0.262; *d* = 1.224), and *Il1*β (FC = 2.612; *p* = 0.268; *d* = 1.048).

*ll12b* (FC = 1.01, *p* = 0.976; *d* = 0.026), *Foxp3* (FC = 0.750; *p* = 0.657; *d* = 0.391), and *ll1r1* (FC = 0.750; *p* = 0.628; *d* = 0.428) were neither significantly upregulated nor downregulated. *Cohen’s d* effect sizes ranged from small to medium.

#### 3.2.3 Anti-inflammatory marker

*Il10* was upregulated without statistical significance (*p* = 0.276), a fold change of 2.502, and a large *Cohen’s d* effect size (*d* = 1.029).

#### 3.2.4 Pro/anti-inflammatory marker

*Ifna1* was neither significantly up-nor downregulated (*p* = 0.829) with a fold change of 0.900 and a small *Cohen’s d* effect size (*d* = 0.188).

## 4 Discussion

This study was the first to characterize *early* vocal deficits in the well-established *TgF344-AD* rat model of Alzheimer’s disease and the first to confirm inflammation and *early* AD-related gene expression dysregulation in the TA muscles of *TgF344-AD* rats for future preclinical investigations and subsequent translation. We hypothesized that progressive deficits in USVs would be found in *TgF344-AD* rats, along with inflammation and AD-related pathology in the TA muscle. The findings of our study allow us to accept these hypotheses. Our data revealed that *TgF344-AD* rats demonstrate changes in mean power of simple and FM vocalizations from 6 to 9 months of age. Moreover, this was not seen in WT rats, supporting that this vocal deficit is vulnerable to the *TgF344-AD* phenotype. A reduction in intensity over time is in line with clinical AD findings of later-stages of the disease. In addition, we found significant downregulation of several AD-related genes in the TA muscle tissue of *TgF344-AD* rats at 12 months.

Vocal deficits and communication dysfunction are highly prevalent in AD (Bayles et al., 2000; Han et al., 2014; Ranasinghe et al., 2017; Mueller et al., 2018a), particularly in the later stages and potentially already long before diagnosis (Meilan et al., 2018; Mueller et al., 2018a,b, 2021; Hajjar et al., 2023). Although existing studies identify differences in vocal behavior between individuals with AD dementia vs. controls, hypotheses are limited to cognitive and affective contributions to these changes, likely due to the advanced stage of disease of the studies’ participants. For example, Ranasinghe et al. (2017) attributed abnormal vocal behavior in pitch to impaired prefrontal modulation of sensorimotor integration and memory deficits, while others attribute changes in vocal expression to affective and emotion regulation deficits (Misiewicz et al., 2018; Heilman and Nadeau, 2022). Very little is known about early vocal signs/symptoms and their progression due to delayed AD-diagnosis and the lack of diagnostic procedures for AD-related early vocal signs. Therefore, studying voice deficits and disease-related laryngeal inflammation in humans is very challenging. As a result, *early-stage* vocal deficits and laryngeal pathology remain vastly understudied, which is unfortunate due to their potential as early biomarkers. Additionally, these significant gaps in knowledge limit our understanding of overall AD pathophysiology and associated peripheral involvement, inflammatory processes, early identification of AD and AD-typical early signs, as well as potential treatment options to improve patient trajectory throughout the disease.

### 4.1 Ultrasonic vocalizations

Prior research has examined various vocal parameters in individuals with AD and healthy controls: Bae and colleagues found that loudness and loudness variability during speech are decreased in AD compared to controls, indicating diminished intensity and monotonous loudness (Bae et al., 2023). Similarly, results from research completed by Meilan and colleagues reveal that individuals with AD diagnosis read a standard passage with lower intensity compared to non-pathological senescence and mild cognitive impairment groups (Meilan et al., 2018). While vocal communication may not be considerably impaired until later stages of the disease, changes to vocal parameters, such as intensity, may serve as a potential behavioral biomarker for *earlier* detection and timely treatment.

As with mean power, *TgF344-AD* rats have altered peak and high frequency over time, while WT rats showed no changes. Interestingly, *TgF344-AD* peak frequency *increased* from 6 to 9 months for simple *and* FM calls. Generally, changes to the voice can occur as a result of aging. Peak frequency, for example, declines with age (Basken et al., 2012). Since *TgF344-AD* rats displayed a positive rather than negative change in peak frequency, and WTs showed no change from 6 to 9 months, the results suggest that this vocal irregularity is most likely not rooted in age-related changes and may be specific to this AD phenotype. Our findings further revealed that WT rats show typical age-dependent decline in average principal and average low frequency of simple calls over time as described in the literature (Basken et al., 2012). This is not significantly different in the age-matched AD rats.

Interestingly, we observed that WT FM calls are longer in 9 months-old rats than WT calls at 6 months, however, we did not find such progression in *TgF344-AD* rats, even though their calls were significantly longer at 9 months compared to baseline WT FM calls. We measured significantly longer simple calls regardless of genotype, which could be a common aging-related effect. In fact, in an aging study by Basken and colleagues, the duration of step up calls (which are a part of the simple call group category in our study) has been described as increased in 32 month-old *Fischer 344/Brown Norway* rats compared to 9 month-old rats of the same genotype (Basken et al., 2012).

Due to the overall low number of harmonic calls regardless of genotype, no interactions or main effects could be determined in this call type category. The low number of harmonic calls (split calls) is a typical phenomenon in ultrasonic investigations regardless of genotype/strain and is therefore often excluded in statistical analyses. In fact, split calls are the least common 50-kHz subtype call with approximately 0.5% of calls in 20,000 calls (Wright et al., 2010).

### 4.2 Inflammation and AD-pathology in the thyroarytenoid

Dysregulation of AD pathology-related genes and dysregulated inflammatory processes are typical indicators for AD pathogenesis and contributors to the progression of AD. In the central nervous system, downregulation of AD-related genes seems to dominate (Blalock et al., 2011). However, altered levels of candidate AD and inflammatory gene biomarkers in the central and peripheral nervous systems have been reported in the literature with differences in type of dysregulation: while some genes happen to be significantly upregulated in one biomarker and/or region, downregulation can occur in other regions, depending on timepoint during disease progression (Park et al., 2020). This is very critical to the TA muscles, particularly considering that a minority of neural tissue can be found in the TA that may be expressing the transgenes initially juxtaposed with normal striated muscle compensation for neural dysregulation.

The results of this study indicated significant *early-stage* downregulation of AD-related genes *Ubiquinol-Cytochrome C Reductase Core Protein 2 (Uqcrc2)*, protein coding gene *Beta-Secretase 2 (Bace2)*, *Serine Peptidase Inhibitor 3 N (Serpina3n),* and *Insulin-like Growth Factor-2 (Igf2)* in the TA muscle tissue of *TgF344-AD* rats at 12 months. Furthermore, we found downregulation of *Plakophilin-4 (Pkp4), Cathepsin-L (Ctsl),* and *Protein Kinase C-delta (Prkcδ)*, however, without reaching the indicated significance threshold of *p* ≤ 0.05. In human early-onset AD brains, *Uqcrc2* is downregulated contributing to mitochondrial dysfunction (Adav et al., 2019). *Bace2* is highly expressed in tracheal tissue (Bennett et al., 2000) and can therefore be seen as an important marker in laryngeal tissue when comparing WT with AD samples. Interestingly, while upregulated in the central nervous system (Holler et al., 2012; Zattoni et al., 2022), our results suggest that *Bace2* and *Serpina3n,* are downregulated in the TA muscle in the *early stage* of the disease. It is important to note that this is just a genetic transcription and does not necessarily indicate corresponding low protein levels. There could be a negative feedback in the setting of the *Tg*-model driving secondary compensatory transcription changes in nearby tissue. *Igf2*, the neurotrophic peptide insulin-like growth factor-2, has neuroprotective function (Beletskiy et al., 2021) and was expectedly downregulated in *TgF344-AD* rats at 12 months. *Pkcδ* is usually upregulated in human AD brains and plays a role in amyloid-β processing and activation of the immune system (Lucke-Wold et al., 2015; Du et al., 2018). A loss of *Ctsl* has been reported to lead to accumulation of amyloid-β precursor protein (APP) and amyloid-β peptides (Cermak et al., 2016). While we found up- and downregulation of specific AD-pathology related genes, typical disease-stage related patterns of gene expressions in the TA muscle remain ambiguous due to the lack of comparative literature, however, our results clearly indicate significant dysregulation of AD-related genes. It is not necessarily surprising to see opposite transcriptional profiles in adjacent tissues (*see*: Blalock et al., 2011); one area might be primarily-affected by transgene expression and the other secondarily-affected. More research expanding on up- and downregulation in various stages during the AD progression is necessary to map typical peripheral dysregulation of these genes.

Anti-inflammatory cytokines such as *Interleukin-10* (*Il-10)* reduce inflammation during AD pathogenesis with increased levels in the central and peripheral nervous systems indicating disease severity with rapid cognitive decline (Strle et al., 2001; Leung et al., 2013; Park et al., 2020). Our results found *Il-10* to be upregulated, however, without reaching statistical significance. This might indicate the beginning of anti-inflammatory responses to *early* AD-related inflammation in the TA muscle given that, although not statistically significant, the mean *Il-10* expression was expressed over 2.5-fold that of WT thyroarytenoid samples, clearly indicating the presence of anti-inflammatory differential gene expression in the TA. With a large effect size *Cohen’s d* value (Table 5), it is likely that undersampling is the reason for a lack of significance. This will be addressed with future work.

**Table 5:** Genes and their respective *Cohen’s d* value to demonstrate effect sizes.

Gene	Cohen’s *d*
*Uqcrc2*	6.712
*Myd88*	2.399
*Bace2*	3.117
*Serpina3n*	2.502
*Igf2*	2.200
*Rorc*	2.572
*Il2*	1.556
*Jun*	1.493
*Prkcδ*	1.449
*Ctsl*	1.360
*Ifnγ*	1.224
*Il1β*	1.048
*Il10*	1.029
*Il1α*	0.975
*Il6*	0.954
*Pkp4*	0.935
*Il1r1*	0.428
*Foxp3*	0.391
*Ifnα1*	0.188
*Il12b*	0.026

Furthermore, our results showed significant downregulation of pro-inflammatory innate immune signal transduction adaptor *Myeloid Differentiation Factor 88 (Myd88)* in *TgF344-AD* rats at 12 months of age. While many genes such as *Interleukin-1 Receptor Type 1* (*Il1r1*) and *Myd88* form proinflammatory signaling cascades and are upregulated in human AD (Zarezadeh Mehrabadi et al., 2022), reduced *Myd88* expression has demonstrated to result in greater cognitive deficits and spatial memory impairment, as well as increased soluble oligomeric amyloid-β in APP_swe_/PS1 transgenic mice, emphasizing the importance in microglia activation to mitigate AD evolution (Michaud et al., 2011). Even though our results did not yield statistical significance for the upregulation of pro-inflammatory markers *Interleukin 1 Beta (Il1β), Interleukin 2 (Il2)*, and *Interleukin 6 (Il6)*, these findings, when seen as trends, are in alignment with current research on upregulation of gene expressions in different peripheral biomarkers (Park et al., 2020). Interestingly, *Interleukin 1 Alpha* (*Il1α*) was downregulated without significance. However, it is notable that some genes in this study (such as *Il6*) were highly upregulated (others, respectively, highly downregulated) resulting in high Standard Error of the Mean (SEM) values, which led for these gene dysregulations to not reach statistical significance. This could be a result of our small sample size. When combined with more data points in the future, we expect these genes to reach statistical significance based on the current literature, our trending findings, and the magnitudes of their effects (Table 5). Additionally, our study revealed upregulation of the pro-inflammatory cytokine *Interferon Gamma (Ifnγ),* even though it did not reach statistical significance. Importantly, contradictory effects through simultaneous disease-promoting and disease-ameliorating functions in relation to certain cytokines (such as *Ifnγ*) and AD-related degeneration have been reported in the literature (Mastrangelo et al., 2009; Park et al., 2020). Therefore, monitoring and characterizing pro-, anti-inflammatory, and other AD pathology-related gene expressions in laryngeal tissues and their respective up-/downregulation during Alzheimer’s disease progression is necessary for 1) a complete understanding of potential dichotomous activities of cytokines, 2) the better understanding of inflammation cascades during different disease stages, and subsequently 3) to improve our understanding of AD pathogenesis and the onset and progression of vocal deficits in the future to facilitate early detection and the development of therapeutic interventions. Importantly, considering that peripheral inflammation differs from CNS inflammation and that there could be dichotomous activities of cytokines, or pleiotropy depending on receptor expression, with differential effects on various monocyte populations in striated muscle (macrophages vs. microglia), certain treatment approaches targeting the CNS might worsen peripheral effects of AD. This should be considered in the development of vocal deficit treatment as well as in overall AD treatment approaches.

### 4.3 Limitations and future directions

This study only included male rats; future work should include female rats as potential sex-based differences emerge during disease progression (Lenell and Johnson, 2017; Demetrius et al., 2021). The timepoints chosen in this study represent two early timepoints (6 and 9 months of age) but do not encompass the entire AD progression, which has been assayed up to 24 months of age in previous studies (Cohen et al., 2013). More research is needed to fully characterize peripheral inflammation in the vocal-related nervous system across all stages of AD in the *TgF344-AD* rat. These data will be essential for understanding pathogenesis and vocal impairments across all stages of AD.

This study suggests that parameters such as vocal intensity (mean power) may be valuable targets for using voice as an early biomarker for clinical AD identification and subsequent treatment. Future research should investigate potential correlations between inflammatory responses/AD-related gene expressions in the larynx and vocal deficits. Additionally, since AD encompasses cognitive and affective systems, we acknowledge that deficits in communication reflect this as well as sensorimotor deficits, as is true with many progressive neurogenic communication disorders such as Parkinson disease (Smith and Caplan, 2018). Due to the presence of microgliosis, astrogliosis, and learning deficits at 6 months of age (Cohen et al., 2013), additional work is necessary to differentiate between strictly motoric and behavioral components of vocalization deficits.

Ultrasonic vocalizations were elicited in a naturalistic setting that used a mating paradigm to evoke calls. A female rat that displayed clear signs of estrus was paired with the experimental male rat and was removed after two mounting attempts. Male-only vocalizations were then recorded while the experimental rat freely moved in their homecage. WT and *TgF344-AD* rats across all timepoints generally displayed a variety of behaviors while vocalizing. We acknowledge that different behaviors (grooming vs. exploration) can elicit distinct call types (Bogacki-Rychlik et al., 2021). In this study, behaviors and acoustic analyses were not specifically time-linked and we are therefore not able to clearly identify underlying behaviors of a call, which could be viewed as a limitation. However, because all rats from both genotypes displayed a variety of behaviors in relatively similar ratios across all testing periods, we view this as a robust and controlled approach to test our hypotheses. This method has been used in our previous work for drug studies, across different disease models, in therapeutic trials, and in aging research (Ciucci et al., 2010; Basken et al., 2012; Johnson et al., 2013; Ringel et al., 2013; Grant et al., 2018; Hoffmeister et al., 2022; Broadfoot et al., 2023; Cullins et al., 2023), and as such we are able to directly compare findings across different models due to similar data collection and analyses.

Furthermore, the small sample size from our pilot tissue for RT-qPCR analysis and additional methodological restraints, such as precluding the use of low-quality RNA resulting in misleading *p*-values, pose limitations that should be considered in future methodological approaches, given that effect size calculations support significance of our findings with a modestly larger sample size (Table 5). We therefore propose to further investigate protein levels via *Western blot* analyses or *enzyme-linked immunosorbent assays* (*ELISA*) in the future to determine if there is mutant human AD protein in the TA muscles, the recurrent laryngeal nerve, and the cranial nerve X (vagus nerve), and to further establish their appearance during the disease progression. Additionally, *Laser Capture Microdissection* and subsequent *RNA sequencing* could be used in future investigations to characterize different expression profiles in the TA muscle and adjacent tissues with improved specificity. Particularly given the differences in tissue composition in the TA muscle (epithelium vs. connective tissue vs. muscle fiber types), this technique could shed light on which specific areas are most affected and which structures particularly contribute to vocal dysfunction. Further investigations will be necessary to disambiguate temporal and spatial relationships of inflammatory and AD-related gene expressions. *Spatial Transcriptomics* could be used to investigate distinct peripheral locations in the larynx at various timepoints during disease progression. Additionally, correlations between inflammation in the CNS and vocal acoustics should be investigated within the same organism.

Lastly, it is noteworthy that two transgenes are expressed under the direction of Prp (APP; PS1) which results in decent CNS specificity but may lose effects in peripheral tissues. Additionally, *Tg* expression might also result in an earlier course of AD than seen in humans.

## 5 Conclusion

This work capitalizes on recent research that converges on the hypotheses that the peripheral nervous system is affected in the prodromal and early stages of AD, and that early voice and communication changes can be used as potential early behavioral biomarkers.

We demonstrated early vocal deficits and confirmed prodromal dysregulation of AD pathology related genes and inflammatory genes in the TA muscle of the *TgF344-AD* rat. Overall, results from this study validate the *TgF344-AD* model for early-stage investigations of vocal dysfunction and its underlying pathological mechanisms for subsequent clinical translation.

Conclusive evidence for early-stage up- vs. downregulation of AD-related gene expressions and inflammation markers in the peripheral nervous system remains limited and more research needs to be conducted to fill gaps in knowledge and to clarify contradictory findings in the literature. Further preclinical investigations are necessary to better understand the various underlying mechanisms of *early* AD-related vocalization changes to inform the development of evidence-based *early* diagnostic and treatment options in humans, and to improve quality of life and overall health in individuals with AD in the future.

## Data availability statement

The original contributions presented in the study are included in the article/supplementary material, further inquiries can be directed to the corresponding author.

## Ethics statement

The animal study was approved by the University of Wisconsin-Madison School of Medicine and Public Health Institutional Animal Care and Use Committee (IACUC) and was performed in accordance with the National Research Council’s Guide for the Care and Use of Laboratory Animals (The National Academies Press, 2011). The study was conducted in accordance with the local legislation and institutional requirements.

## Author contributions

DR: Conceptualization, Data curation, Formal analysis, Investigation, Methodology, Visualization, Writing – original draft, Writing – review & editing. MK: Data curation, Visualization, Writing – original draft, Writing – review & editing. DB: Data curation, Formal analysis, Investigation, Methodology, Visualization, Writing – original draft. KM: Writing – original draft, Writing – review & editing. JR: Conceptualization, Funding acquisition, Resources, Validation, Writing – review & editing. NC: Conceptualization, Funding acquisition, Methodology, Project administration, Resources, Supervision, Validation, Writing – review & editing. MC: Conceptualization, Funding acquisition, Methodology, Project administration, Resources, Supervision, Validation, Writing – review & editing.
